# Characterization of a Chickpea Mutant Resistant to *Phelipanche aegyptiaca* Pers. and *Orobanche crenata* Forsk

**DOI:** 10.3390/plants10122552

**Published:** 2021-11-23

**Authors:** Shmuel Galili, Joseph Hershenhorn, Evgeny Smirnov, Koichi Yoneyama, Xiaonan Xie, Orit Amir-Segev, Aharon Bellalou, Evgenia Dor

**Affiliations:** 1 Institute of Plant Sciences, The Volcani Center, Agricultural Research Organization, P.O. Box 15159, HaMaccabim Road 68, Rishon LeZion 7505101, Israel; oritas@volcani.agri.gov.il (O.A.-S.); aharonb@volcani.agri.gov.il (A.B.); 2 Institute of Plant Protection, Newe Ya’ar Research Center, Agricultural Research Organization, P.O. Box 1021, Ramat Yishay 3009503, Israel; hershenj@gmail.com (J.H.); evgeny@volcani.agri.gov.il (E.S.); 3 Center for Bioscience Research and Education, Utsunomiya University, 350 Mine-machi, Utsunomiya 321-8505, Japan; yone2000@sirius.ocn.ne.jp (K.Y.); xie@cc.utsunomiya-u.ac.jp (X.X.)

**Keywords:** chickpea, strigolactone, broomrape resistance, chickpea phenotype, chlorophyll, carotenoid, anthocyanin

## Abstract

Chickpea (*Cicer arietinum* L.) is a major pulse crop in Israel grown on about 3000 ha spread, from the Upper Galilee in the north to the North-Negev desert in the south. In the last few years, there has been a gradual increase in broomrape infestation in chickpea fields in all regions of Israel. Resistant chickpea cultivars would be simple and effective solution to control broomrape. Thus, to develop resistant cultivars we screened an ethyl methanesulfonate (EMS) mutant population of F01 variety (Kabuli type) for broomrape resistance. One of the mutant lines (CCD7M14) was found to be highly resistant to both *Phelipanche aegyptiaca* and *Orobanche crenata.* The resistance mechanism is based on the inability of the mutant to produce strigolactones (SLs)—stimulants of broomrape seed germination. LC/MS/MS analysis revealed the SLs orobanchol, orobanchyl acetate, and didehydroorobanchol in root exudates of the wild type, but no SLs could be detected in the root exudates of CCD7M14. Sequence analyses revealed a point mutation (G-to-A transition at nucleotide position 210) in the Carotenoid Cleavage Dioxygenase 7 (CCD7) gene that is responsible for the production of key enzymes in the biosynthesis of SLs. This nonsense mutation resulted in a CCD7 stop codon at position 70 of the protein. The influences of the CCD7M14 mutation on chickpea phenotype and chlorophyll, carotenoid, and anthocyanin content were characterized.

## 1. Introduction

Chickpea (*Cicer arietinum* L.) is an important legume crop grown on over 10 million ha in at least 37 countries worldwide, including India (65%), Pakistan (10%), Iran (8%), and Turkey (5.5%). [1]. In Israel chickpea is one of the main legume crops, grown on about 3000 ha with an average yield of about 3.5 t/ha. In recent years, the two broomrape species in Israel, Egyptian broomrape (*Phelipanche aegyptiaca* Pers.) and crenate broomrape (*Orobanche crenata* Forsk.), have become a major problem in chickpea field production [2]. The only broomrape-control methods that have been successfully utilized commercially in other crops are resistant varieties and chemical control [3,4,5].

Broomrapes (*Phelipanche* spp. and *Orobanche* spp.) are worldwide weedy root parasites of dicotyledonous crops, causing severe losses in the yield and quality of agricultural crops [6,7]. The initial step of broomrape–plant recognition involves root-exuded strigolactones (SLs), which have long been known to induce broomrape seed germination [8,9], and have been recently recognized as plant hormones affecting plant development and growth [10]. SLs consist of a tricyclic lactone (A, B, and C rings) connected to a butenolide group (D ring) via an enol ether bridge. SLs’ degree of activity, function, and specificity depend on the various substituents on the A and B rings [11]. The SL biosynthesis pathway in plants is derived from the carotenoid pathway [12,13,14], in which β-carotene is converted into carlactone by three catalytic enzymes: D-27 (9-cis/all-*trans*-β-carotene isomerase) [15], and two carotenoid cleavage dioxygenases, CCD7 and CCD8 [16,17]. Carlactone is converted to SLs by the cytochrome P450 monooxygenase- homolog activity of MORE AXILLARY GROWTH1 (MAX1) in rice [18], and MAX1 and lateral branching oxidoreductase in *Arabidopsis* [16,19,20]. SLs are produced mainly in roots and their active transport to the rhizosphere by the exporter pleiotropic drug resistance 1 (PDR1), identified in *Petunia*, was shown [21,22,23].

In a previous study, we obtained a tomato CCD7-deletion mutant showing broomrape resistance [24,25]. SL-deficient sorghum and rice mutants also demonstrate high degrees of resistance to *Striga* spp. [26,27]. Moreover, resistance to parasitic weeds based on low SL exudation exists in pea and faba bean germplasms [28,29]. Mutants defective in SL biosynthesis are characterized by a highly branched/tillering phenotype [30,31]. Furthermore, SLs regulate root architecture [16,32,33,34,35].

The objectives of the present study were to isolate and characterize an ethyl methanesulfonate (EMS)-mutagenized F01 chickpea mutant, CCD7M14, which shows considerable resistance to broomrape, and to elucidate its resistance mechanism, characterize its phenotype, and determine its leaf chlorophyll, carotenoid and anthocyanin contents.

## 2. Results

### 2.1. Mutagenesis and Screening for Broomrape Resistance

EMS mutagenesis was applied to seeds of a wild-type (WT) F01 chickpea breeding line (Kabuli type), and 3000 families of the second generation were tested for resistance to both *P. aegyptiaca* and *O. crenata*. A chickpea mutant showing high resistance to both broomrapes, was identified—CCD7M14 (Figure S1).

### 2.2. Phenotyping

#### 2.2.1. Resistance to *P. aegyptiaca* and *O. crenata*

**Figure 1:**
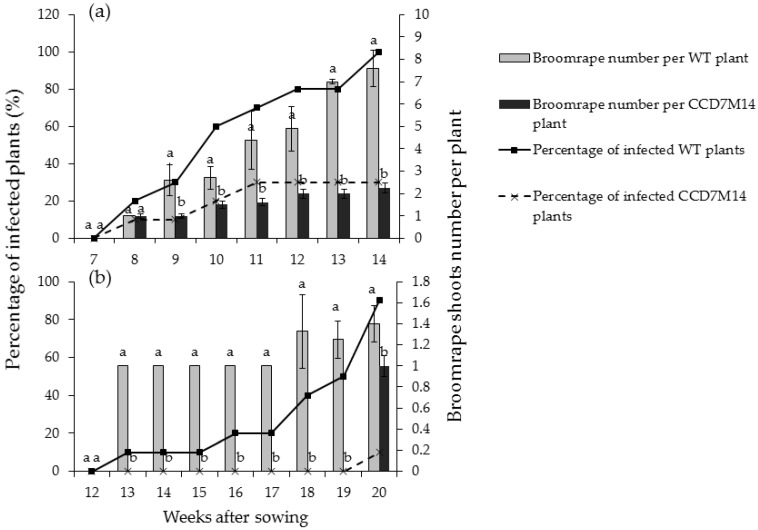
Aboveground broomrape shoots in pots planted with WT F01 or the CCD7M14 mutant. The experiments were arranged in a completely randomized design with 10 replications (pots) per treatment. Lines show the percentages of infected plants, bars indicate the average numbers of aboveground shoots attached to the infected plants. (**a**) Infestation with *P. aegyptiaca*. (**b**) Infestation with *O. crenata.* Vertical lines indicate standard error of the mean (SEM). Lowercase letters indicate least-significant differences (LSD), based on the Tukey–Kramer honestly significant difference test (α = 0.05) between the chickpea lines.

*P. aegyptiaca* shoots began to emerge aboveground 8 weeks after sowing in pots with WT F01 plants. At this time, about 20% of the WT F01 plants were infected with one or two shoots (Figure 1a). Both shoot number above the soil and percentage of infected plants increased rapidly over time, and at the end of the experiment (14 weeks), all WT F01 plants were infected with 8–10 aboveground shoots. At this time only one broomrape shoot was observed in two pots planted with CCD7M14 (percentage of infected plants was 20%). Throughout the course of the experiment, both percentage of infected plants and number of aboveground shoots per plant were significantly lower for the mutant plants. The roots were washed and broomrape number and biomass were recorded. About 16.10 ± 4.23 broomrape shoots were counted per WT F01 plant with average biomass of 82.11 ± 6.69 g, whereas only 1.60 ± 1.78 shoots with total biomass of 7.93 ± 5.15 g were found per mutant plant (Table 1).

*O. crenata* developed more slowly than *P. aegyptiaca*. First *O. crenata* shoots emerged aboveground 12 weeks after planting in WT F01 pots (Figure 1b). At the end of the experiment (20 weeks after sowing), 90% of WT F01 plants were infected with one or two shoots. About 13.6 ± 3.48 broomrapes with a total biomass of about 109.74 ± 10.92 g per WT F01 plant were observed after root washing (Table 1). CCD7M14 plants were highly resistant to *O. crenata.* Only one aboveground shoot was observed in one pot at the end of the experiment, and about 2.20 ± 1.71 broomrapes with a total biomass of 13.78 ± 6.96 g were counted on the washed roots (Table 1).

#### 2.2.2. Resistance Mechanism

To determine whether the resistance mechanism of CCD7M14 was based on its inability to synthesize SLs or secrete them into the rhizosphere, we tested its ability to stimulate broomrape seed germination. *P. aegyptiaca* seed germination near the WT F01 root system was high (76.84 ± 6.28%), whereas in the pots with CCD7M14 plants, only 0.72 ± 0.45% of the seeds germinated. Germination of *O. crenata* seeds was about 42.12 ± 2.57% in the pots with the WT, whereas in the mutant pots, no *O. crenata* seed germination was observed (Table 2).

The ability of WT and CCDM14 root exudates to stimulate *P. aegyptiaca* seed germination was tested in vitro in Petri dishes. Root exudate of the WT applied to the seeds at concentrations of 0.1, 1 and 10 μL/mL caused *P. aegyptiaca* germination at rates of 28.1 ± 5.78, 77.38 ± 3.13, and 84.84 ± 4.28%, respectively (compared to 79.19 ± 1.7% following application of 10^−6^ M GR24, a synthetic SL, as a positive control) (Table 3). A low percentage of seed germination was induced by the mutant root exudates (9.02 ± 0.77, 15.94 ± 1.19, and 34.95 ± 2.52% at concentrations of 0.1, 1 and 10 μL/mL, respectively), but only short radicals developed, which did not continue to elongate normally and were dead after 1 week.

Analysis of SLs in root exudates of WT F01 and CCD7M14 plants revealed the presence of orobanchol, orobanchyl acetate, and putative didehydroorobanchol in WT F01 root exudates, but no SLs in the mutant root exudates (Figure S2).

#### 2.2.3. Plant Morphology and Pigment Contents

**Figure 2:**
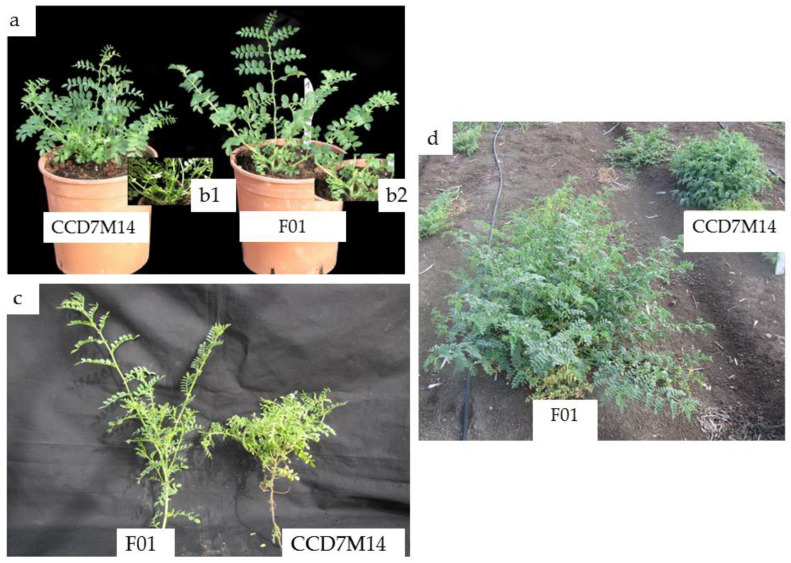
Morphological differences between WT F01 and CCD7M14. (**a**) One-month-old WT F01 (right) and CCD7M14 (left) plants grown in a net house. (**b1**,**b2**) Stem distribution on the lower section of the plants. (**c**) Primary branches of WT F01 (left) and CCD7M14 (right) plants. (**d**) Three-month-old WT F01 (left) and CCD7M14 (right) plants in the field.

CCD7M14 plants had a SL-deficiency phenotype, with a high number of short branches compared to WT F01 plants. No significant differences in foliage or root biomass were found between the lines (Table 4). The CCD7M14 plants had 83% more primary branches than the WT F01 plants, and the mutant’s primary branch length was only 66% of that of the WT F01 plant. These morphological changes in CCD7M14 were observed both in the net house and under field conditions (Figure 2a–d), leading to a bushy shape at plant maturity.

Analysis of carotenoid, chlorophyll, and anthocyanin contents in the first, third, and fifth leaves revealed significant decreases in carotenoids and chlorophylls, and an increase in anthocyanins in the mutant as compared to its parental line (Table 5).

### 2.3. DNA Analysis

**Figure 3:**
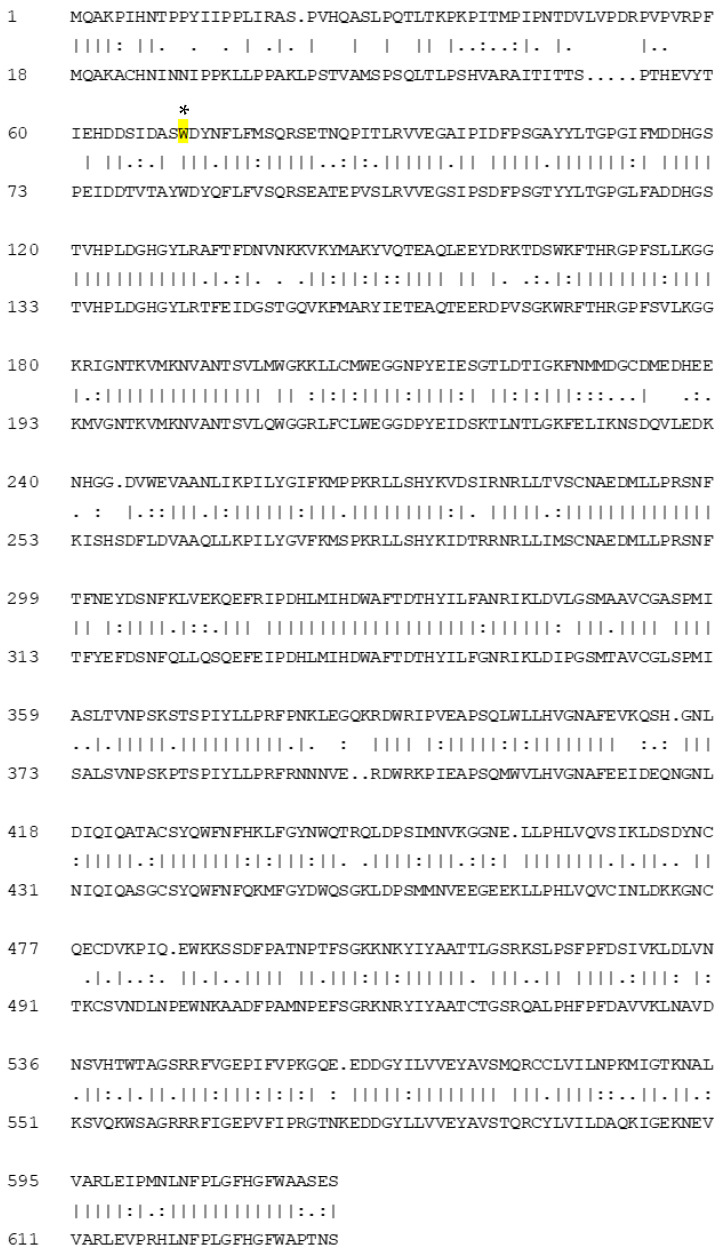
Chickpea and tomato CCD7 protein sequence homology. The upper and lower sequences are of chickpea and tomato CCD7, respectively. Identical amino acids are indicated by a solid line, and similar amino acids are indicated by one or two dots according to their similarity levels. The mutated amino acid in CCD7 of broomrape-resistant line CCD7M14, at position 70 (84 according to tomato) is indicated in yellow and marked by an asterisk.

**Figure 4:**
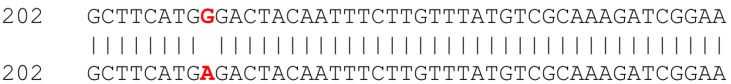
The Blast results of DNA sequences (nucleotides 202–246) of WT F01 chickpea (upper line) and CCD7M14 (lower line). The G-to-A transition at position 210 is indicated in bold red letters.

**Figure 5:**
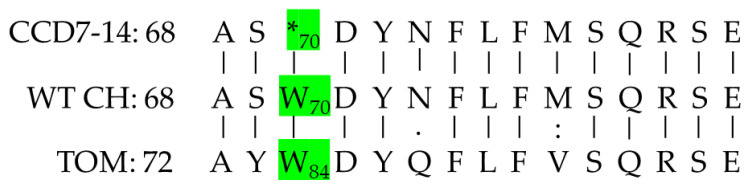
The Blast results of the protein sequences (amino acids 68–82 (82–96 in tomato)) of CCD7M14 (CCD7-14), F01 (WT CH), and tomato (TOM). The W-to-stop codon (*) transition at position 70 (84 in tomato) is indicated in green.

Blast analyses of the chickpea genome based on the tomato *CCD7* sequence revealed a single *CCD7* gene with 64.9% protein sequence identity to the tomato protein (Figure 3). DNA sequence analysis of the *CCD7* gene in CCD7M14 compared to the WT F01 line revealed a single G-to-A nucleotide transition at position 210 (Figure 4). This mutation led to stop-codon formation (*) instead of tryptophan (W) at amino acid position 70 (84 in tomato) (Figure 5). No other mutations were found in the chickpea *CCD7* gene.

## 3. Discussion

Chickpea mutant CCD7M14 was produced by EMS mutagenesis. The mutant showed high resistance to both *P. aegyptiaca* and *O. crenata* (Figure S1). Only one mutant plant was infected with a single *P. aegyptiaca*, and one with a single *O. crenata* shoots in all experiments, compared to 90–100% infection in WT F01 plants with more than 8–10 aboveground broomrape shoots (Figure 1a,b). However, once an attachment formed on the mutant roots, parasite development progressed normally. Since no *P. aegyptiaca* or *O. crenata* seed germination was observed near CCD7M14 roots, and its root exudates did not stimulate their seed germination in Petri dishes, it is suggested that the CCD7M14 resistance mechanism is based on its inability to synthesize SLs or to secrete them into the rhizosphere. Indeed, DNA sequence analysis of the CCD7M14 *CCD7* gene revealed stop-codon formation due to a single G-to-A nucleotide transition at position 210 (Figure 4 and Figure 5). This resulted in the absence of the SLs orobanchol, orobanchyl acetate, and didehydroorobanchol in the root exudates (Figure S2), rendering the mutant plant resistant to the parasite because no seed germination could occur near its roots. This resistance mechanism has been reported in tomato [25,36,37,38], pea [39] and faba bean [30,40]. Previously, this type of resistance had been obtained by fast-neutron mutagenesis [24,25] and targeted mutagenesis [37,38]. It had also been found in wild tomato species (*Solanum pennellii* [36]), and recognized in resistant cultivars of faba bean and pea [30,39,40]. In our case, the resistance was obtained by EMS mutagenesis, where one point mutation in the *CCD7* gene resulted in the formation of a stop codon, leading to the same results as *CCD7* deletion by fast-neutron mutagenesis [25,26] or silencing of *CCD8* using CRISPR/Cas9-mediated mutagenesis [37,38]. It is important to note that to date, all identified *CCD7* genes have been single copies, in contrast to two, four and six copies of *CCD8* identified in maize, rice and sorghum, respectively [41].

It has been shown that plants exude mixtures of several SLs, and every plant species is characterized by a specific SL profile [42]. In the current study, we first identified the SLs produced by chickpea roots. LC/MS/MS analysis revealed that the WT F01 chickpea cultivar produces three SLs: orobanchol, orobanchyl acetate, and putative didehydroorobanchol isomer(s). All three belong to the orobanchol type, which only differs from the strigol-type SLs in the stereochemistry of the C-ring [43], and are derived from 4-deoxyorobanchol in rice [18]. Some other species, such as *Populus*, pea, petunia, and tomato, have been reported to have only orobanchol-type SLs [44]. Orobanchol, first isolated from red clover (*Trifolium pratense* L.) root exudates [9], is probably the most abundant hydroxy-SL in the plant kingdom [42]. This SL assumes to be a central intermediate in SL biosynthesis, and it has been suggested as a precursor of other SL molecules, such as: fabacol, orobanchyl acetate, solanacol, and so on [43]. Putative didehydroorobanchol has been detected in root exudates of tomato [26], tobacco [42], and *Medicago truncatula* [45]; and orobanchyl acetate in red clover [46], rice and tobacco [47]. The didehydroorobanchol isomer in *M. truncatula* was named medicaol [45]. Both orobanchol and orobanchyl acetate have been reported to be produced by Asteraceae plants and by faba bean [48,49].

CCD7M14 was characterized by a typical SL-deficient phenotype—increased branching and reduced plant height. These results are in agreement with Vogel et al. [50], where transgenic tomato plants expressing their endogenous *CCD7* gene in the antisense form also displayed increased branching and reduced plant height. Similar observations have also been reported for pea [51], petunia [52], poplar [53], and *Arabidopsis* [54]. According to Boyer et al. [11], orobanchyl acetate and 5- deoxystrigol are more active at inhibiting shoot branching than strigol and orobanchol. Furthermore, blockage of orobanchol biosynthesis from carlactonoic acid in tomato did not rescue the branching phenotype [55]. The absence of orobanchyl acetate in CCD7M14 plants likely explains its bushy shape at maturity (Figure 5). Orobanchol and putative didehydroorobanchol may be involved in other biological processes, such as regulation of photosynthesis and pigment accumulation. We found significant decreases in chlorophyll and carotenoid contents and an increase in anthocyanins in the leaves of CCD7M14 as compared to the WT F01 line (Table 2). Exogenous application of the synthetic SL GR24 under stress conditions has been shown to control chlorophyll degradation and maintain the photosynthetic rate [56,57,58,59]. On the other hand, chlorophyll content in sunflower leaves was not influenced by GR24 treatment of achene pre-sowing, but carotenoid content increased [60]. GR24 has been found to affect ABA-induced activation of anthocyanin biosynthesis in grapevine berries [61]. In transgenic tobacco lines impaired in SL biosynthesis, overaccumulation of anthocyanins in the mature stems likely results from antagonism between the SL and jasmonic acid pathways [62]. SL regulation of anthocyanin accumulation has been shown in *Arabidopsis* [63,64].

## 4. Materials and Methods

### 4.1. Plant Material

All experiments were carried out with: (a) a WT F01 chickpea breeding line (Kabuli type) that is erect, produces high yields, and is resistant to both *Fusarium* wilt and *Ascochyta* blight and (b) CCD7M14, a chickpea EMS mutant line derived from WT F01. Broomrape seeds were collected from *P. aegyptiaca* and *O. crenata* inflorescences parasitizing tomato plants grown in Kibbutz Bet Ha’shita (32°33′15″ N 35°26′15″ E) and chickpea plants grown in Kibbutz Kfar H’horesh (32°42′7.56″ N 35°16′27.47″ E), respectively. The inflorescences were dried at 23–35 °C for 2 months and then the seeds were separated with a 300-mesh size sieve (50 µm) and stored in the dark at 4 °C until use.

### 4.2. Mutagenesis

WT F01 chickpea breeding line seeds were used for mutagenesis. Approximately 6000 WT F01 seeds were allowed to swell in water for 10 h and then exposed to the mutation inducer EMS at a concentration of 4% (vol/vol) which, according to the dose-response curve, decreased seed germination by 50%. After shaking at 50 rpm for 10 h, the EMS was removed, and the seeds were washed under running tap water for 14 h. The seeds were dried under airflow for 48 h and delivered to Shorashim Nursery Ltd., Israel, to produce seedlings. The seedlings were planted and grown in a field at the Western Galilee experimental farm, Israel (32°55′ N 35°04′ E), to produce M_2_ seeds.

### 4.3. Screening for Broomrape Resistance

An EMS-mutated population of about 3000 families (each derived from a single M_1_ plant) was used to screen for broomrape resistance. Eight M_2_ generation seeds from each family were seeded separately in soil containing seeds of *P. aegyptiaca* and *O. crenata* at a concentration of 20 mg seeds per kg of soil (~3000 seeds/kg). After 3 months, plant roots were evaluated for broomrape infection. Families of plants that were free of broomrape were selected for the next screening, leading to identification of the broomrape-resistant mutant CCD7M14.

### 4.4. Phenotype Determination

#### 4.4.1. Evaluation of Broomrape Resistance

Broomrape-resistance tests were conducted in 2 L pots, each filled with soil mixed with the seeds of *P. aegyptiaca* and *O. crenata* at a concentration of 20 mg seeds per kg soil. Control pots did not contain broomrape seeds. Each pot was planted with one chickpea plant. Organic medium-heavy clay–loam soil collected in Newe Ya’ar Research Center (32°42′9″ N, 35°10′9″ E) was used in all experiments. The plants were grown in nethouse and irrigated and fertilized as needed. The experiments were arranged in a completely randomized design with 10 replications (pots) per treatment. Once a week, the number of broomrape shoots per pot was evaluated. At the end of the experiments, the roots were gently washed out of the pots under tap water and broomrape number and fresh biomass were determined.

#### 4.4.2. Resistance Mechanism Determination

The ability of WT and mutant plants to induce germination of *P. aegyptiaca* and *O. crenata* seeds was tested in GF/A glass microfiber filter paper envelopes [25]. Briefly, *P. aegyptiaca* or *O. crenata* seeds inside the paper envelopes were placed close to the chickpea roots at planting. Seed germination percentage was recorded four weeks after planting using a stereoscopic microscope. Control pots (without plants) were used for spontaneous seed germination determination.

To analyze SLs in root exudates, WT F01 and CCD7M14 plants were grown under hydroponic conditions with feeding solution circulated through activated charcoal [25]. Once a week, the charcoal was washed with water and extracted with acetone. The acetone solutions were combined and evaporated under reduced pressure at 35 °C (Rotavapor, Büchi, Switzerland) from all samples. The residue was dissolved in 200 mL water and the solution was extracted three times with equal volumes of ethyl acetate. The ethyl acetate fractions were combined, washed with 0.2 M K_2_HPO_4_ (pH 8.3), dried over anhydrous Na_2_SO_4_, and concentrated under reduced pressure at 35 °C. Dry extracts were stored at 4 °C.

Samples of root exudates were tested for the ability to germinate preconditioned *P. aegyptiaca* seeds according to Yoneyama et al. (2007) [65]. Briefly, dried root exudates were dissolved in methanol up to concentration of 0.2, 2 and 20 μg/mL of which 100 μL was applied to filter paper inside 45-mm diameter Petri dishes. After drying under air flour, 0.2 mL of sterile water was added to the disks to get final concentrations of 0.1, 1, and 10 μg/mL. Disinfected *P. aegyptiaca* seeds were distributed on a 45 mm filter paper disk and kept moistened for 1 week. Then the disks with seeds on them were dried gently on sterile filter paper and transferred to the Petri dishes upon the disks containing root exudates. For the positive control, stimulation with GR24 at a concentration of 10^−6^ M was used. The plates were kept at 25 °C for 10 days, and the *P. aegyptiaca* seed germination was evaluated utilizing of a stereoscopic microscope.

LC-MS/MS analysis of proton adduct ions of SLs was performed with a triple quadrupole/linear ion trap instrument (LIT) (QTRAP5500; AB Sciex) with an electrospray source according to Yoneyama et al., 2007 [65]. All peaks corresponding to strigolactones were confirmed by *P. aegyptiaca* seed-germination assay [25].

#### 4.4.3. Plant Morphology and Pigment Contents

The plants of WT F01 and CCD7M14 were grown in 4 L pots in Newe Ya’ar organic soil. After 14 weeks, the plants were harvested by cutting the stems at the pot’s soil surface. First, third and fifth leaves were sampled for determination of total carotenoid, anthocyanin, and chlorophyll a and b contents. The number of primary branches, the number of secondary branches per primary branch, and foliage and root fresh biomass were determined.

Contents of carotenoids and anthocyanin were measured according to Segev et al. [66], and chlorophyll were was measured according to Lichtenthaler [67]. Briefly, chlorophyll and anthocyanin were extracted using methanol and acidic methanol (99% methanol and 1% hydrochloric acid), respectively. The test tubes were incubated at room temperature for two days in the dark. After two days, the solutions were tested in a spectrophotometer at 665, 652, 530, and 470 nm wavelengths. From the data we calculated the relative amounts of total chlorophyll, chlorophyll a, chlorophyll b, total carotenoids, and total anthocyanins according to the following formulas:

Chlorophyll A (μg/mL) = 16.72 × A_665_ − 9.16 × A_652_; Chlorophyll B (μg/mL) = 34.09 × A_652_ − 15.28 × A_665_; Total chlorophyll (a + b) (μg/mL) = 1.44 × A_665_ + 24.93 × A_652_; Total carotenoids (μg/mL) = (1000 × A_470_ − 1.63 × Chlorophyll A − 104.96 × Chlorophyll B)/221; Total anthocyanins (μg/mL) = (449.1 × A_530_ + 24.93 × 2000)/24,500.

The final results were calculated in μg per 1 g of fresh leaf biomass.

### 4.5. DNA Extraction and PCR Amplification

Total genomic DNA was extracted from young leaves of 2-week-old M_3_ plants homozygous for broomrape resistance. Primer design, PCR amplification, electrophoresis in a 1.0% agarose gel, and sequence analysis of the *CCD7* gene were performed as described by Schreiber et al. [68], with several modifications: annealing was performed at 55 °C for 30 s and synthesis at 72 °C for 60 s. Eight pairs of primers, purchased from Syntezza Bioscience Ltd. (Jerusalem, Israel), were used (Table 6).

### 4.6. Statistical Analysis

All experimental results were subjected to ANOVA utilizing JMP software, version 5.0 (SAS Institute Inc., Cary, NC, USA). Data on seed germination were separated by standard error of the mean (SEM). To meet the assumption of ANOVA, percentage data were arcsine-transformed before analysis. The results on the number of aboveground broomrape shoots were compared by SEM and by least-significant differences (LSD), based on Tukey–Kramer honestly significant difference test (α = 0.05). Data on the number and biomass of *P. aegyptiaca* and *O. crenata* attached to chickpea roots after root washing were separated by SEM. The experiments on chickpea lines sensitivity to *P. aegyptiaca* and *O. crenata* were repeated twice. The repeated experiments were compared using Fisher’s *t*-test, which showed homogeneity of variances; therefore, the data were combined. The test of the differences in morphology between WT F01 and CCD7M14 and the data of pigment concentration in chickpea leaves was conducted with 5 and 3 replicates, respectively and separated by SEM. The results were analyzed by LS means contrast test (α = 0.05).

## 5. Conclusions

Using EMS mutagenesis, chickpea line CCD7M14 showing high resistance to both *O. crenata* and *P. aegyptiaca* was developed. The resistance mechanism was based on blockage of SL synthesis, probably caused by stop codon formation due to the point mutation in the *CCD7* gene. Root exudates of the mutant did not contain SLs. The mutant plants displayed increased branching and reduced plant height; decreased chlorophyll and carotenoid contents; and increased accumulation of anthocyanin in the leaves compared with the WT.

## Figures and Tables

**Table 1:** Statistical analysis of the chickpea resistance experiments. The results were subjected to ANOVA. The experiments were conducted with ten replications. SEM—standard error of the mean, dF—Degrees of Freedom; F—F ratio, Prob > F—F probability.

Parameter	Chickpea Line	Average Mean	SEM	dF	F	Prob > F
*P. aegyptiaca*						
Broomrape number	WT F01	16.1	4.23	1	99.96	<0.0001
	CCD7M14	1.6	1.78			
Broomrape biomass (g)	WT F01	82.11	6.69	1	77.17	<0.0001
	CCD7M14	7.93	5.15			
*O. crenata*						
Broomrape number	WT F01	13.6	3.48	1	9.6	0.0062
	CCD7M14	2.2	1.71			
Broomrape biomass (g)	WT F01	109.74	10.92	1	54.92	<0.0001
	CCD7M14	13.78	6.96			

**Table 2:** Statistical analysis of the broomrape seed germination closed to chickpea roots. The results were subjected to ANOVA. The experiments were conducted with five replications. SEM—standard error of the mean, dF—Degrees of Freedom; F—F ratio, Prob > F—F probability.

Broomrape	Chickpea Line	Average Mean	SEM	dF	F	Prob > F
*P. aegyptiaca*	WT F01	76.84	6.28	1	146.00	<0.0001
	CCD7M14	0.72	0.45			
*O. crenata*	WT F01	42.21	2.57	1	270.07	<0.0001
	CCD7M14	0	0			

**Table 3:** Statistical analysis of the broomrape seed germination caused by root exudates. The results were subjected to ANOVA. The experiments were conducted with five replications. SEM—standard error of the mean, dF—Degrees of Freedom; F—F ratio, Prob > F—F probability.

Root Exudates Concentration (μL/mL)	Chickpea Line	Average Mean	SEM	dF	F	Prob > F
1	WT F01	28.10	5.78	1	10.71	0.0307
	CCD7M14	9.02	0.77			
10	WT F01	77.38	3.13	1	336.71	<0.0001
	CCD7M14	15.94	1.19			
100	WT F01	84.84	4.28	1	100.95	0.0006
	CCD7M14	34.95	2.52			

**Table 4:** Morphological characteristics of CCD7M14 compared to WT F01 chickpea.

Parameters	Chickpea Line	
	WT F01	CCD7M14
Foliage biomass (g)	242.8 ± 14.5 a	210.2 ± 12.5 a
Root biomass (g)	112.3 ± 8.3 a	113.8 ± 37.2 a
Primary branch number	7.0 ± 0.8 b	12.0 ± 1.4 a
Primary branch length (cm)	62.6 ± 2.0 a	40.3 ± 4.0 b

Data are presented as average mean of 5 replications with standard error of the mean (SEM). Lowercase letters indicate significant differences between the WT F01 and CCD7M14 according to LS means contrast test (α = 0.05).

**Table 5:** Contents of carotenoids, chlorophyll, and anthocyanins (μg per 1 g of fresh leaf biomass) in the leaves of WT F01 and CCD7M14.

Pigment	Leaf 1		Leaf 3		Leaf 5	
	WT F01	CCD7M14	WT F01	CCD7M14	WT F01	CCD7M14
Chlorophyll a	214.5 ± 9.2 a	120.3 ± 15.1 b	230.0 ± 30.0 a	157.2 ± 9.5 b	281.5 ± 48.9 a	151.1 ± 30.6 b
Chlorophyll b	183.9 ± 7.4 a	56.0 ± 11.7 b	184.3 ± 47.9 a	74.5 ± 5.0 b	200.0 ± 35.3 a	65.2 ± 17.2 b
Total chlorophyll	402.2 ± 11.5 a	176.3 ± 26.5 b	414.43 ± 80.5 a	231.7 ± 6.1 b	481.6 ± 56.7 a	216.3 ± 53.2 b
Carotenoids	62.1 ± 5.3 a	35.1 ± 4.0 b	66.5 ± 12.1 a	35.8 ± 2.8 b	61.3 ± 8.1 a	27.2 ± 4.5 b
Anthocyanin	9.7 ± 0.9 b	33.2 ± 5.1 a	15.2 ± 2.1 b	32.6 ± 7.3 a	11.9 ± 1.2 b	29.3 ± 4.2 a

Results are presented as average mean of 3 replications with standard error of the mean (SEM). Lowercase letters indicate significant differences between the WT F01 and CCD7M14 according to LS means contrast test (α = 0.05).

**Table 6:** Primer sets used in this study.

Primer Set	Exon	Forward Primer	Reverse Primer	Product Size (bp)	Sequenced Region (cDNA)
1	1	AGCACATTTTGTTGCCAAGC	TCCTGCTTACATGAAATGCAAACT	1090	1–529
2	1	GAGTACGATCGAAAGACTGACTCG	TCCTGCTTACATGAAATGCAAACT	551	522–776
3	2	TACAAGGTGTACAACATTGAGT	ACTGCCAATTTGTTGGCATTTC	599	777–908
4	3	GAAATGCCAACAAATTGGCAGT	GCATGCTTAAATTTCATTTTGGA	621	909–1043
5	4	TCATGAGGGAGTAAATAATCAACA	TTTAATTCACGTTTTATGTCGGT	623	1044–1316
6	5	AGGGACAAAAATTATCGGCTT	CTTAGGATAAACCACACATAGATAG	361	1317–1404
7	6	CCAATTAAGATGTTCGAGAGCT	ACATGGACAAATCTATAACGACA	747	1405–1710
8	7	AGTAATAGCTAATCAAAACGGGT	TTGGATTTCCAAGAGTCCAAT	686	1711–1872

## Data Availability

Datais contained within the article and Supplementary Material.

## Supplementary Materials

The following are available online at https://www.mdpi.com/article/10.3390/plants10122552/s1, Figure S1: WT F01 (right) and CCD7M14 (left) plants growing in soil mixed with seeds of *P. aegyptiaca* at a concentration of 20 mg seeds per kg soil. Figure S2: Selected reaction monitoring (SRM) chromatograms of WT F01 (**a**) and CCD7M14 (**b**) root exudates. Determination of SLs was based on the retention time and transition of *m*/*z* 345 > 97 for didehydroorobanchol and 347 > 233 for orobanchol and orobanchyl acetate.
