# Supplementary material for: Characterization of a Chickpea Mutant Resistant to Phelipanche aegyptiaca Pers. and Orobanche crenata Forsk
Source: Plants (Basel). 2021 Nov 23;10(12):2552. doi: 10.3390/plants10122552 (PMC8705912; doi:10.3390/plants10122552)
Supplement: Supplementary file 1 [file plants-10-02552-s001.zip › plants-1362278-supplementary.pdf]

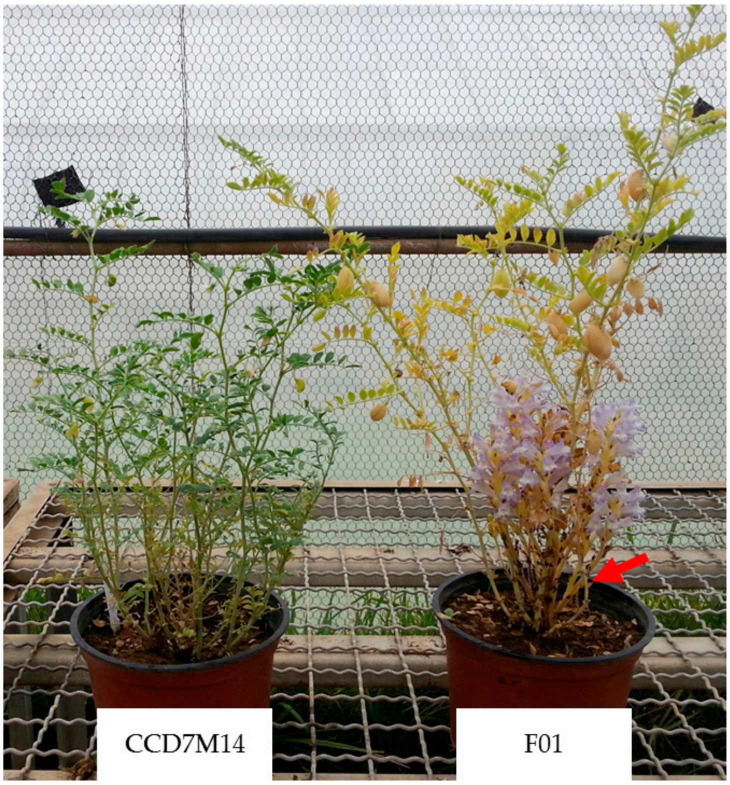

Figure S1. WT F01 (right) and CCD7M14 (left) plants growing in soil mixed with seeds of *P. aegyptiaca* at a concentration of 20 mg seeds per kg soil.

a)

b)

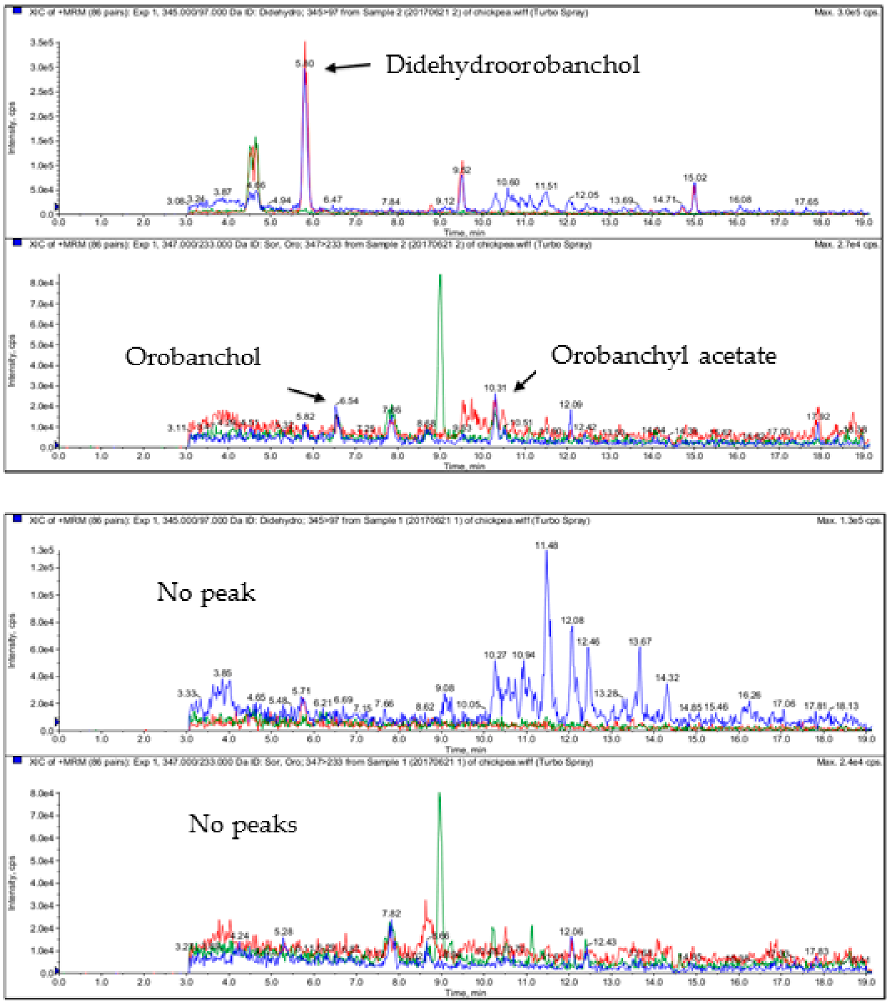

Figure S2. Selected reaction monitoring (SRM) chromatograms of WT F01 (**a**) and CCD7M14 (**b**) root exudates. Determination of SLs was based on the retention time and transition of *m/z* 345 > 97 for didehydroorobanchol and 347 > 233 for orobanchol and orobanchyl acetate.
